# Exploring the acute effects of running on cerebral blood flow and food cue reactivity in healthy young men using functional magnetic resonance imaging

**DOI:** 10.1002/hbm.26314

**Published:** 2023-05-05

**Authors:** Alice E. Thackray, Elanor C. Hinton, Turki M. Alanazi, Abdulrahman M. Dera, Kyoko Fujihara, Julian P. Hamilton‐Shield, James A. King, Fiona E. Lithander, Masashi Miyashita, Julie Thompson, Paul S. Morgan, Melanie J. Davies, David J. Stensel

**Affiliations:** ^1^ National Centre for Sport and Exercise Medicine, School of Sport, Exercise and Health Sciences, Loughborough University Loughborough UK; ^2^ National Institute for Health and Care Research (NIHR) Leicester Biomedical Research Centre, University Hospitals of Leicester NHS Trust and University of Leicester Leicester UK; ^3^ National Institute for Health and Care Research (NIHR) Bristol Biomedical Research Centre Nutrition Theme, University of Bristol Bristol UK; ^4^ Department of Respiratory Therapy College of Applied Medical Sciences, King Saud bin Abdulaziz University for Health Sciences Al Ahsa Saudi Arabia; ^5^ King Abdullah International Medical Research Center Al Ahsa Saudi Arabia; ^6^ College of Sport Sciences, Jeddah University Saudi Arabia; ^7^ Graduate School of Sport Sciences, Waseda University Tokorozawa Japan; ^8^ Liggins Institute, University of Auckland Auckland New Zealand; ^9^ Department of Nutrition and Dietetics, Faculty of Medical and Health Sciences, University of Auckland Auckland New Zealand; ^10^ Faculty of Sport Sciences Waseda University Tokorozawa Japan; ^11^ University Hospitals of Leicester NHS Trust, Infirmary Square Leicester UK; ^12^ Radiological Sciences School of Medicine, University of Nottingham UK; ^13^ National Institute for Health and Care Research (NIHR) Nottingham Biomedical Research Centre Nottingham UK; ^14^ Diabetes Research Centre, University of Leicester Leicester UK; ^15^ Department of Sports Science and Physical Education The Chinese University of Hong Kong, Ma Liu Shui Hong Kong

**Keywords:** appetite, brain, cerebral blood flow, exercise, fMRI, food cue reactivity

## Abstract

Acute exercise suppresses appetite and alters food‐cue reactivity, but the extent exercise‐induced changes in cerebral blood flow (CBF) influences the blood‐oxygen‐level‐dependent (BOLD) signal during appetite‐related paradigms is not known. This study examined the impact of acute running on visual food‐cue reactivity and explored whether such responses are influenced by CBF variability. In a randomised crossover design, 23 men (mean ± SD: 24 ± 4 years, 22.9 ± 2.1 kg/m^2^) completed fMRI scans before and after 60 min of running (68% ± 3% peak oxygen uptake) or rest (control). Five‐minute pseudo‐continuous arterial spin labelling fMRI scans were conducted for CBF assessment before and at four consecutive repeat acquisitions after exercise/rest. BOLD‐fMRI was acquired during a food‐cue reactivity task before and 28 min after exercise/rest. Food‐cue reactivity analysis was performed with and without CBF adjustment. Subjective appetite ratings were assessed before, during and after exercise/rest. Exercise CBF was higher in grey matter, the posterior insula and in the region of the amygdala/hippocampus, and lower in the medial orbitofrontal cortex and dorsal striatum than control (main effect trial *p* ≤ .018). No time‐by‐trial interactions for CBF were identified (*p* ≥ .087). Exercise induced moderate‐to‐large reductions in subjective appetite ratings (Cohen's *d* = 0.53–0.84; *p* ≤ .024) and increased food‐cue reactivity in the paracingulate gyrus, hippocampus, precuneous cortex, frontal pole and posterior cingulate gyrus. Accounting for CBF variability did not markedly alter detection of exercise‐induced BOLD signal changes. Acute running evoked overall changes in CBF that were not time dependent and increased food‐cue reactivity in regions implicated in attention, anticipation of reward, and episodic memory independent of CBF.

## Graphical abstract

We investigated the impact of a single running bout on cerebral blood flow (CBF) and food cue reactivity using functional magnetic resonance imaging (fMRI) in healthy men. Running did not alter the time‐course of grey matter or regional CBF up to 30 min after exercise cessation but increased food cue reactivity in brain regions implicated in attention, reward and episodic memory retrieval. Exercise‐related BOLD signal changes were detected in the presence of underlying CBF.

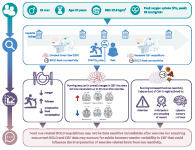

## 1 INTRODUCTION

The interaction of exercise with appetite control and food intake attracts widespread interest due to the ability of exercise to influence weight management (Blundell et al., 2015; Donnelly et al., 2009). Consistent evidence demonstrates that single exercise bouts transiently suppress appetite and the absence of energy intake compensation in the subsequent hours preserves the exercise‐evoked energy deficit in the short term (Dorling et al., 2018; Schubert et al., 2013). Food intake regulation is influenced by hedonic systems in the brain that are sensitive to internal metabolic signals and the external food environment (Berthoud et al., 2017). Although understanding of the intricate brain networks that control eating behaviour has evolved in recent decades (Watts et al., 2022), the impact of exercise on the neural circuitry underpinning hedonic drivers of eating is not well characterized.

Appetite‐related brain responses can be explored using blood‐oxygen‐level‐dependent (BOLD) functional magnetic resonance imaging (fMRI), which is often performed during visual food cue reactivity tasks (Dagher, 2012). Such paradigms have been shown to activate brain regions associated with hedonic, motivational and sensory responses including the insula, basal ganglia, orbitofrontal cortex (OFC), hippocampus and visual cortex (Huerta et al., 2014; Tang et al., 2012; van der Laan et al., 2011; van Meer et al., 2015). An ongoing systematic review (PROSPERO CRD42020193938) has identified limited evidence which indicates that single exercise bouts alter brain responses during food cue reactivity tasks, with lower responsiveness to food cues observed after exercise in brain regions linked to food reward and motivation including the insula (Evero et al., 2012), OFC (Crabtree et al., 2014), hippocampus (Crabtree et al., 2014) and putamen (Evero et al., 2012). However, the evidence is sparse and not consistent with greater reactivity to food cues, particularly of low hedonic value, also reported after acute exercise in the insula and putamen (Crabtree et al., 2014), and exercise‐induced alterations in brain responses to food cues are not supported universally (Saanijoki et al., 2018). Further neuroimaging studies are required to better understand how exercise influences neural responses to visual food stimuli. This should include investigations that account for baseline variability between exercise and control trials given current evidence is restricted to comparisons from fMRI scans conducted after equivalent periods of exercise and rest.

Exploring immediate exercise‐induced effects on food cue reactivity could be influenced by the dynamic cardio‐pulmonary changes that alter cerebrovascular function in response to an exercise bout. The elevated brain neural activity and metabolic demands of exercise are met by a concomitant increase in cerebral blood flow (CBF) up to intensities of ~60% of maximum oxygen uptake beyond which CBF typically declines towards baseline values (Ogoh & Ainslie, 2009). Alterations in CBF have also been observed immediately after exercise including in studies utilizing arterial spin labelling (ASL) MRI which permits non‐invasive quantification of CBF by magnetically labelling arterial blood entering brain tissue (Alsop et al., 2015). Specifically, reductions in grey matter and hippocampus CBF have been reported when measured up to 12 min after exercise (Mast et al., 2022; Olivo et al., 2021), although exercise‐mediated increases in whole brain CBF have also been identified (Smith et al., 2010).

Investigations of the time‐course of exercise‐induced changes in regional CBF have demonstrated both increased (Steventon et al., 2020) and decreased (MacIntosh et al., 2014) CBF in the hippocampus when measured in repeated post‐exercise scans. The reported perturbations in hippocampus CBF persisted in the final ASL acquisition at 40–60 min after exercise cessation, whereas grey matter CBF appeared relatively stable in the immediate post‐exercise period (MacIntosh et al., 2014; Steventon et al., 2020). However, these studies are limited by the absence of a resting control trial, and the time‐course of CBF changes after exercise for applications related to appetite have yet to be established. This is important for determining the optimum time to acquire food cue reactivity BOLD data after exercise in addition to establishing the extent that post‐exercise CBF influences food cue stimulated neural activity.

The primary aims of this study were to (i) track the temporal pattern of CBF in the immediate post‐exercise period to identify the optimum time to conduct fMRI acquisitions using a food cue paradigm and (ii) determine the importance of accounting for CBF effects that may influence the BOLD signal during a food cue reactivity task. As a secondary aim, we sought to characterize the effect of acute exercise on neural responses to food cues varying in energy density. It was hypothesized that exercise would increase grey matter and regional CBF compared to a resting control trial, but the changes would show a time‐dependent effect, returning to baseline within 30 min of exercise cessation.

## 2 METHODS

### 2.1 Ethical approval and participant eligibility

The study received approval from Loughborough University's Research Ethics Sub‐Committee. Twenty‐three healthy men provided written informed consent to participate in the study. Recruitment was restricted to men as previous work has identified sex‐based differences in neural responses to visual food cues particularly under fasted conditions (Chao et al., 2017). Participants were eligible if they were aged 18–45 years, non‐smokers, reported being weight stable (≤3 kg change in the previous 3 months) and had no known cardiovascular or metabolic diseases. Participants had no contraindications to MRI scanning, were not dieting or taking any medication, and habitually consumed a Western European or Mediterranean style diet (confirmed verbally during screening and after inspection of diet records completed before the main trials). Participant characteristics are displayed in Table 1.

**TABLE 1:** Participant characteristics.

Age (years)	24 ± 4
Stature (m)	1.77 ± 0.06
Body mass (kg)	72.0 ± 8.9
Body mass index (kg/m^2^)	22.9 ± 2.1
Waist circumference (cm)	76.9 ± 5.9
Body fat (%)	14.2 ± 5.8
Lean body mass (kg)	61.4 ± 5.3
Peak oxygen uptake (mL/kg/min)	63 ± 6
Dominant hand (right/left)	20/3
*International Physical Activity Questionnaire*	
Total physical activity (MET‐min/week)	3337 ± 1514
*Three factor eating questionnaire*	
Dietary restraint	7 ± 4
Dietary disinhibition	6 ± 2
Hunger	7 ± 3

*Note*: Values are mean ± SD for *n* = 23. Body fat percentage was estimated using bioelectrical impedance analysis.

Abbreviation: MET, metabolic equivalent.

### 2.2 Preliminary measures

Participants were screened to determine eligibility for the study and completed questionnaires assessing general health status, MRI safety, habitual physical activity levels (short form International Physical Activity Questionnaire; Craig et al., 2003) and eating behaviour traits (Three Factor Eating Questionnaire; Stunkard & Messick, 1985). Measurements of stature and body mass were recorded (Seca 285, Seca GmbH & Co.KG, Germany) and waist circumference was quantified at the narrowest point of the torso between the lower rib margin and the iliac crest. Participants performed submaximal incremental and peak oxygen uptake treadmill (Technogym ExciteMed, Cesena, Italy) tests as described previously (Alotaibi et al., 2021). Exhaled gas samples were measured continuously using an online breath‐by‐breath gas analysis system (Metalyser 3B, Cortex, Biophysik, Germany), and heart rate was monitored throughout using short‐range telemetry (Polar A3, Kempele, Finland).

### 2.3 Experimental design

**FIGURE 1:**
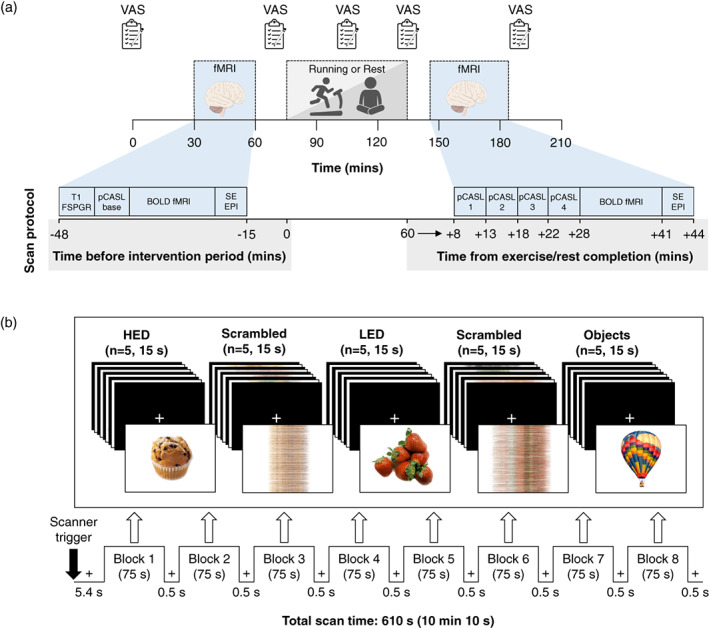
Schematic of the study protocol. (a) Timeline of the trial procedures and functional magnetic resonance imaging (fMRI) scan protocol. Scans were conducted before and after 60 min of running and rest and involved iterations of the following sequences: (1) 3D sagittal T1‐weighted fast spoiled gradient echo (FSPGR) anatomical; (2) pseudo‐continuous arterial spin labelling (pCASL); (3) task‐based blood‐oxygen‐level‐dependent (BOLD) fMRI; and (4) spin‐echo echo‐planar imaging (SE EPI). Ratings of perceived appetite were measured using visual analogue scales (VAS). (b) Block design of the task‐based BOLD fMRI scan. Participants viewed eight blocks containing visually matched images of high‐ and very high‐energy density foods (HED) (total *n* = 40), very low‐ and low‐energy density foods (LED) (total *n* = 40), non‐food objects (total *n* = 40) and scrambled images of the two food categories (total *n* = 80). Images were presented in a random order for 2.5 s with a 0.5 s inter‐stimulus interval when a central fixation cross was displayed. Images were obtained from a freely available database (Blechert et al., 2014, 2019).

Using a within‐measures, crossover design, participants completed two experimental trials in a random order separated by at least 1 week: (1) exercise and (2) control. The study design is presented in Figure 1a. Participants weighed and recorded all food and drink consumed in the 24 h before the first experimental trial and replicated this dietary pattern before the subsequent experimental trial. Participants were instructed to avoid caffeine, alcohol and strenuous physical activity during the 24 h standardization period before both trials. A standardized meal consisting of a margherita pizza was consumed in the evening before the two experimental trials (4452 kJ, 50.4% carbohydrate, 32.1% fat, 17.5% protein). No additional food or drink items, except plain water, were permitted at the meal or before arrival at the laboratory the next morning. Adherence to the dietary and physical activity requirements during the standardization period was confirmed verbally upon attendance at the laboratory.

### 2.4 Main trials

Participants were instructed to consume 250 mL of plain water before arrival at the laboratory between 08:00 and 08:30 having fasted overnight for at least 12 h. Body mass and body fat percentage were measured at the start of each trial using bioelectrical impedance analysis (Seca Ltd, Hamburg Germany). After 20 min of seated rest, participants underwent a baseline fMRI scan for the assessment of CBF and food cue reactivity. Baseline fMRI scans were staggered to start at either 08:45 or 09:30 and participants were scanned in identical time slots for both trials. After the baseline fMRI scan, participants rested in a semi‐supine position for 60 min in the control trial or ran for 60 min at ~70% of their peak oxygen uptake during the exercise trial. Heart rate was monitored continuously during exercise, and breath‐by‐breath exhaled gas samples were measured in both trials to calculate the net energy expenditure and substrate oxidation during exercise (Frayn, 1983). The treadmill speed was adjusted periodically during exercise to ensure the target exercise intensity was achieved. Participants underwent a second fMRI scan for repeated assessments of CBF and food cue reactivity which commenced, on average, 8 ± 3 min (mean ± SD) after completing the 60 min exercise or rest periods.

### 2.5 Ratings of perceived appetite

Subjective ratings of hunger, fullness, satisfaction and prospective food consumption were recorded using 100 mm visual analogue scales (Flint et al., 2000) at 0 min (baseline), 70 min (pre‐exercise/rest), 105 min (mid‐exercise/rest), 135 min (post‐exercise/rest), and 190 min (post fMRI). The scales were anchored at 0 and 100 mm by descriptors signifying the extremes of the appetite construct being measured. Additional constructs relating to mood, stress and arousal were integrated to blind participants to the outcomes of interest.

### 2.6 Food cue paradigm

During the food cue reactivity task, four categories of colour images were presented in a single run using a block design: (1) very low‐ and low‐energy density foods (LED; *n* = 40) (e.g., fruits, vegetables, salad); (2) high‐ and very high‐energy density foods (HED; *n* = 40) (e.g., chocolate, cake, nuts); (3) non‐food objects (*n* = 40) (e.g., furniture, stationary, flowers); and (4) scrambled images of the two food categories (*n* = 80). Images were obtained from a freely available database (Blechert et al., 2014, 2019) and were matched between categories for colour, object size, brightness, contrast and complexity (Tables S1 and S2). Images were selected for inclusion based on familiarity (determined by the research team) and food images were restricted, where possible, to single items consumed in a typical Western diet with an equal split of sweet and savoury items assigned to the LED and HED categories. Nutritional information provided in the image database was used to calculate the energy density of food items and a threshold of ≤1.5 and ≥2.26 kcal/g was applied to classify LED (range: 0.09–1.40 kcal/g) and HED (range: 3.11–7.41 kcal/g) foods, respectively (Vernarelli et al., 2018). In line with previous investigations, scrambled images of each food item provided a low‐level baseline that enabled control of colour and visual features but were not identifiable as the food item (English et al., 2016; Goldstone et al., 2014).

Stimuli were presented in eight blocks each containing five images from the LED, HED and non‐food object categories, which were interspersed with 16 blocks each comprised of five scrambled images (Figure 1b). Four versions of the paradigm were developed with two pseudo‐randomized block orders and two orders of image position (left or right of screen) and were assigned in a counterbalanced order across participants. Every image was displayed once per scan session in a random order within each block and was presented for 2.5 s with a 0.5 s inter‐stimulus interval when a central fixation cross was shown. The total time of the food cue paradigm was 10 min 10 s.

Participants viewed the stimulus via a mirror attached to the head coil on a MR‐compatible monitor at the head of the scanner. Visual stimulus presentation and synchronization to fMRI acquisition was controlled using the software package Presentation® version 21.1 (Neurobehavioral Systems, Inc., California, USA). Images were marginally offset from the centre of the screen and participants used a hand‐held button box connected to a Lumina LSC‐400 controller (Cedrus Corporation, California, USA) to indicate left or right position to ensure they were attentive during the task. At the end of the study, food images were rated for familiarity, consumption and liking, and object images were rated for familiarity, using an online survey (supplementary results, Section 2.1).

### 2.7 fMRI data acquisition

Structural and functional MRI was performed on a GE 3.0 T Discovery MR750w scanner (General Electric, Boston, USA) using a 32‐channel head coil. A timeline of the scan protocol is shown in Figure 1a. A 3D sagittal T1‐weighted fast spoiled gradient echo anatomical image was obtained in the baseline scan of the exercise and control trials. Details of the sequence parameters were as follows: TE = 3.1 ms; flip angle = 8°; FOV 240 mm; voxel size = 1 × 1 × 1 mm; slice thickness = 1 mm; scan duration = 4 min 48 s. Perfusion imaging with the product pseudo‐continuous ASL (pCASL) sequence was performed in a single acquisition at baseline in both trials, and during four consecutive repeat acquisitions after completing the 60 min exercise and rest periods. Acquisition parameters were as follows: TR = 4769 ms; TE 10.7 ms; FOV = 240 mm; slice thickness = 4 mm; post label delay = 2025 ms; scan duration = 4 min 23 s. Task‐based fMRI data were acquired in a single acquisition during all four sessions using a non‐product simultaneous multi‐slice (SMS) echo planar imaging (EPI) sequence with the following parameters: TR = 1800 ms; TE = 35 ms; flip angle = 70°; FOV = 211 mm; voxel size 2.2 × 2.2 × 2.2 mm; SMS factor = 2; number of slices = 44 axial; slice thickness = 2 mm; between‐slice gap = 0.2 mm; number of volumes = 360; scan duration = 10 min 48 s. Two spin‐echo EPI volumes with reversed phase‐encoding directions (posterior > anterior and anterior > posterior) were acquired at the end of each scan session for distortion correction: TR = 3800 ms; TE = 42 ms; flip angle = 90°; FOV = 211 mm; voxel size = 2.2 × 2.2 × 2.2 mm; number of slices = 44 axial; slice thickness = 2 mm; between‐slice gap = 0.2 mm.

### 2.8 fMRI data analysis

Analysis of functional data (pCASL and task based) was conducted in the FMRIB Software Library (FSL) version 6.0.4 (Jenkinson et al., 2012; Smith et al., 2004). Image analysis for pCASL data was performed using the CBF map generated by the MRI scanner during data acquisition.

#### 2.8.1 Pre‐processing stages and regions of interest

Detailed information on pre‐processing of functional data is presented in the supplementary methods (Section 1.1). Seven regions of interest (ROIs) were identified a priori which have previously been implicated in responding to visual food cues: amygdala, hippocampus, hypothalamus, insula, nucleus accumbens, OFC and striatum (Huerta et al., 2014; Killgore et al., 2003; Tang et al., 2012; van der Laan et al., 2011; van Meer et al., 2015). Bilateral ROI masks were created by thresholding the anatomical regions defined in the Harvard–Oxford Cortical and Subcortical structural atlases in FSLeyes. The exception was the hypothalamus which was drawn by hand based on the Atlas of the Human Brain (Mai et al., 2008).

#### 2.8.2 Main analysis

##### Pseudo‐continuous arterial spin labelling

Using a non‐parametric permutation approach in FSL's Randomise (Winkler et al., 2014), CBF in grey matter and the seven ROIs identified previously were analysed in two stages:

1. a *baseline analysis* examining between‐trial differences in CBF at baseline using a paired‐sample *t*‐test; and
2. the *primary analysis* examining between‐trial differences in CBF over time using a 2 × 5 (condition × time‐point) ANOVA.

Separate models were run for each grey matter and ROI mask using threshold‐free cluster enhancement (TFCE) and a family‐wise corrected *p* value of *p* < .05. A Bonferroni correction was applied to account for multiple ROI comparisons. Information pertaining to the peak of each activated cluster was extracted using FSL's cluster command after masking the raw stats image with the activated clusters from the corrected TFCE stats image. Mean CBF in grey matter and the ROI masks was extracted using fslstats.

##### Task‐based fMRI

In whole‐brain analysis at the first level, explanatory variables were entered into a general linear model in FSL's fMRI Expert Analysis Tool (FEAT) for the three image categories (LED, HED and non‐food cues) with a separate model run for each scan session per participant. Three contrasts were defined to examine responses to food cues minus non‐food objects ([i] food [HED + LED] > non‐food cues; [ii] HED > non‐food cues; and [iii] LED > non‐food cues) and a further two contrasts were defined to compare responses between high‐ and low‐value foods ([iv] HED > LED; and [v] LED > HED). The first three volumes (5.4 s) of the functional data were discarded to account for saturation effects. Clusters of activation were identified after thresholding the z‐statistic images at z > 3.1 using a corrected cluster significance threshold of *p* < .05.

The contrasts of parameter estimates generated at the first level were used to conduct higher‐level group analysis on the five contrasts in four stages:

1. a *preliminary baseline analysis* (higher level, fixed effects) explored food cue reactivity during the first exposure to the food cue paradigm (either the control or exercise baseline scan depending on randomization) (data presented in the supplementary results, Section 2.2.1);
2. a *comparative baseline analysis* (higher level, fixed effects) examining the effect of trial (exercise > control and control > exercise) on food cue reactivity at baseline;
3. the *primary analysis* compared the pre‐to‐post change in food cue reactivity between the trials (exercise > control and control > exercise) using a higher‐level paired group analysis (mixed analysis, FMRIBs Local Analysis of Mixed Effects (FLAME) 1 + 2); and
4. a *secondary sub‐analysis* restricted the between‐trial comparison to the post‐exercise and post‐rest scan to facilitate comparison with previous literature adopting an equivalent analysis approach (data presented in the supplementary results, Section 2.2.4).

Analysis was performed both with and without adjustment for CBF by adding the appropriate CBF map as a voxelwise (confound) explanatory variable in the higher‐level FEAT model. Information on the peak value of activated clusters was obtained using the atlasquery tool in FSL (and the associated autoaq script), specifying the Harvard–Oxford cortical and subcortical atlases. The FSL tool FEATquery was used to extract the percentage change in BOLD signal for the peak of each activated cluster.

##### Regions of interest analysis

For the primary analysis, pre‐to‐post difference images for the exercise and control trials were created from the first level contrasts of parameter estimates to generate the input images. Paired *t*‐tests were conducted in Randomise to compare between‐trial differences in food cue reactivity in each ROI mask using TFCE and a family‐wise error corrected *p* value of *p* < .05. A Bonferroni correction was applied to account for multiple ROI comparisons. FSL's cluster command was used to derive information on the peak of activated clusters, and the BOLD signal change was extracted using FEATquery.

In all functional analysis (pCASL and task‐based fMRI), only clusters comprising ≥10 continuous voxels are reported. In sensitivity models, all functional data were re‐analysed excluding participants who were left hand dominant (*n* = 3) which did not alter the interpretation of the data (data not shown).

### 2.9 Statistical analyses

The model residuals of the appetite perceptions were shown to follow a Gaussian distribution after inspection using histograms and are presented as mean ± SD unless indicated otherwise. Time‐averaged (per hour) area under the curve (AUC) values for each rating of perceived appetite were computed using the trapezium rule for the pre‐intervention (0–70 min) and intervention (70–190 min) periods. Between‐trial differences in time‐averaged intervention AUC for appetite perceptions were analysed with a linear mixed‐effects model using the *nlme* package in R (version 4.2.0). Models for each appetite perception included trial (exercise versus control) as a fixed effect, participant as a random effect and were adjusted for pre‐intervention AUC to account for baseline differences before exercise/rest commenced. The 95% confidence intervals (CIs) were calculated for mean pairwise differences between experimental trials. Absolute standardised effect sizes (Cohen's *d*) were calculated by dividing the mean difference (exercise versus control) by the pooled SD and thresholds of 0.2, 0.5 and 0.8 were adopted to denote small, medium and large effects, respectively (Cohen, 1988). Interpretation of the data is based on the 95% CI and effect sizes rather than more conventional dichotomous hypothesis testing (Wasserstein et al., 2019).

In exploratory analyses, Pearson's product moment correlation coefficients or Spearman's rank correlation coefficients were calculated to explore associations of between‐trial changes in CBF (pCASL) or BOLD signal (task‐based fMRI) with the exercise net energy expenditure and between‐trial differences in appetite perceptions. Time‐averaged (per hour) total AUC (0–190 min) for appetite perceptions was used in the correlational analysis to incorporate responses during the entire study period (baseline, pre fMRI scan, intervention period and post‐fMRI scan). Correlations for pCASL were performed after calculating a session average for CBF in the exercise and control trials, and correlations for task‐based fMRI were conducted using the BOLD signal change in the peak voxel for activated clusters in the CBF‐adjusted primary analysis (whole brain only).

## 3 RESULTS

### 3.1 Exercise responses

Treadmill running was performed at an average speed of 11.6 ± 1.4 km/h which elicited a mean heart rate of 169 ± 14 beats/min and a mean oxygen consumption of 3.08 ± 0.39 L/min equivalent to 68% ± 3% of peak oxygen uptake. The non‐protein respiratory exchange ratio was 0.93 ± 0.03, which corresponded to a proportional energy contribution of 80% ± 10% carbohydrate and 20% ± 10% fat (assuming zero contribution from protein). The estimated net oxidation of carbohydrate and fat were 174 ± 35 g and 19 ± 9 g, respectively, with an estimated net energy expenditure of 3.62 ± 0.53 MJ.

### 3.2 Ratings of perceived appetite

**FIGURE 2:**
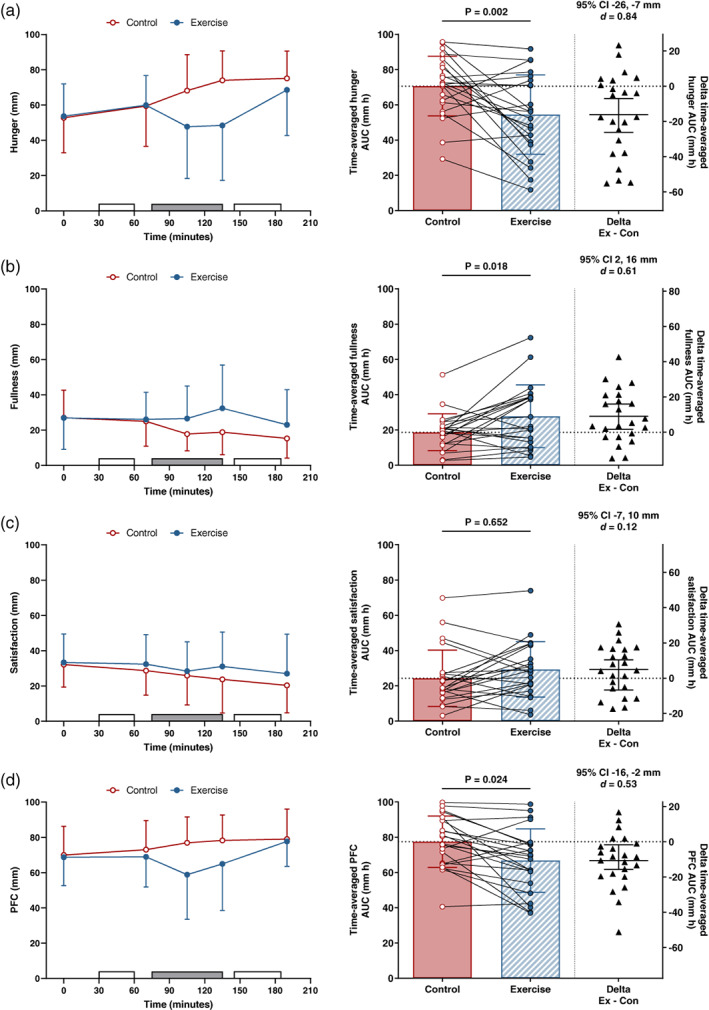
Ratings of perceived (a) hunger, (b) fullness, (c) satisfaction and (d) prospective food consumption (PFC) in the control and exercise trials (*n* = 23). On left‐hand panels, data presented as mean ± SD, open rectangles indicate functional magnetic resonance imaging scan and grey rectangle denotes the 60 min exercise or rest period. On right‐hand panels, the left‐hand y axis displays the time‐averaged area under the curve (AUC) for the intervention period (70–190 min) in the control and exercise trial. Bars and error bars represent the mean ± SD and circles with connecting lines show the individual participant data values. The right‐hand y axis displays the between‐trial difference (exercise minus control) in appetite. Horizontal lines represent the mean difference (95% confidence interval [CI] of the mean absolute difference) and the triangles indicate the between‐trial difference for each participant. For each rating, the *p* value, 95% CI of the mean difference and standardized effect size (Cohen's *d*) is presented for the main effect of trial which was adjusted for the pre‐intervention AUC (0–70 min).

Time‐averaged intervention AUC for appetite perceptions (Figure 2) was lower in the exercise than control trial for hunger (mean difference [95% CI]: −17 [−26, −7] mm h, *d* = 0.84) and prospective food consumption (−9 [−16, −2] mm h, *d* = 0.53). Time‐averaged intervention AUC for fullness was higher in the exercise than control trial (9 [2, 16] mm h, *d* = 0.61). The 95% CI for the mean between‐trial difference in time‐averaged intervention AUC for satisfaction overlapped zero and the standardised effect size was trivial (2 [−7, 10] mm h, *d* = 0.12).

### 3.3 Grey matter and regional CBF using pCASL

#### 3.3.1 Baseline analysis

Grey matter and regional CBF was not statistically different between the exercise and control trial at baseline (all *p* ≥ .077).

#### 3.3.2 Primary analysis

**FIGURE 3:**
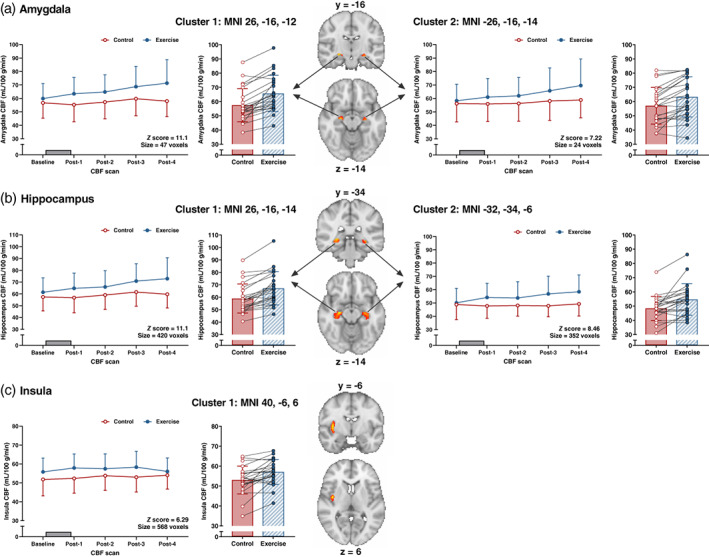
Differences in regional cerebral blood flow (CBF) measured using pseudo‐continuous arterial spin labelling (pCASL) in the (a) amygdala, (b) hippocampus and (c) insula between the exercise and control trial (*n* = 23). Data analysed with a 2 × 5 (condition × time‐point) ANOVA in Randomise (threshold‐free cluster enhancement, family‐wise corrected *p* value of *p* < .05). Brain maps are presented for the main effect of trial representing an average of the five pCASL scans in each trial and are shown in radiological convention with the right hemisphere shown on the left. Red‐yellow clusters represent voxel clusters showing higher CBF in exercise versus control (*p* ≤ .001). Panels adjacent to brain maps depict the extracted CBF data values for the peak of each identified cluster. Data plotted for each CBF scan presented as mean ± SD and grey rectangle denotes the 60 min exercise or rest period. Bar plots represent the mean ± SD for the average of the five pCASL scans in each trial and circles with connecting lines show the individual participant data values. Brain co‐ordinates are presented in MNI space for the peak statistical voxel. MNI, Montreal Neurological Institute.

**FIGURE 4:**
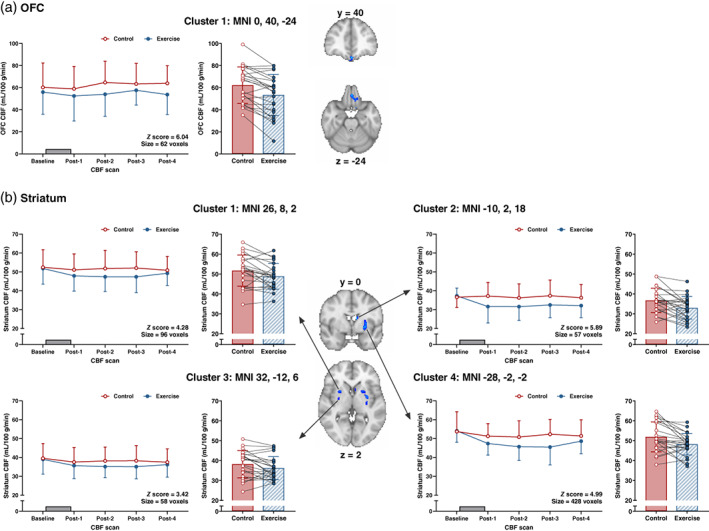
Differences in regional cerebral blood flow (CBF) measured using pseudo‐continuous arterial spin labelling (pCASL) in the (a) orbitofrontal cortex (OFC) and (b) striatum between the exercise and control trial (*n* = 23). Data analysed with a 2 × 5 (condition × time‐point) ANOVA in Randomise (threshold‐free cluster enhancement, family‐wise corrected *p* value of *p* < .05). Brain maps are presented for the main effect of trial representing an average of the five pCASL scans in each trial and are shown in radiological convention with the right hemisphere shown on the left. Blue clusters represent voxel clusters showing higher CBF in control versus exercise (*p* ≤ .018). Panels adjacent to brain maps depict the extracted CBF data values for the peak of each identified cluster. Data plotted for each CBF scan presented as mean ± SD and grey rectangle denotes the 60 min exercise or rest period. Bar plots represent the mean ± SD for the average of the five pCASL scans in each trial and circles with connecting lines show the individual participant data values. Brain co‐ordinates are presented in MNI space for the peak statistical voxel. MNI, Montreal Neurological Institute.

Main effects of trial identified voxel clusters with greater CBF in the exercise than control trial in grey matter, bilaterally in the region of the amygdala/hippocampus and in the right posterior insula (all *p* ≤ .001) (Figure 3). Regional CBF in the left medial OFC and bilateral dorsal striatum (caudate nucleus and putamen) was lower in the exercise than control trial (both *p* ≤ .018) (Figure 4). No trial‐by‐time interactions were identified in grey matter or any of the a priori ROIs (all *p* ≥ .087).

### 3.4 CBF‐adjusted BOLD response for food cue task

#### 3.4.1 CBF‐adjusted baseline comparison (whole brain only)

##### Whole‐brain analysis

Activation in the left frontal pole and right central opercular cortex was lower at baseline in the exercise than control trial in response to food (HED + LED) versus non‐food cues and LED versus non‐food cues (Table 2). Reactivity to HED versus LED cues and LED versus HED cues in the right frontal pole was higher and lower, respectively, in the exercise than control trial at baseline (Table 2).

**TABLE 2:** CBF‐adjusted whole‐brain analysis results of voxel clusters activated in the baseline difference between the exercise and control trials in each contrast (*CBF‐adjusted comparative baseline analysis*).

Contrast	Brain region	Hemisphere	No. of voxels	MNI brain coordinates			*z* value	BOLD signal change (%)	
				*x*	*y*	*z*		Control (baseline)	Exercise (baseline)
***Food (HED + LED) > non‐food***									
Con baseline > Ex baseline	Frontal pole	Left	117	−34	46	10	4.49	0.02 ± 0.15	−0.10 ± 0.23
	Central opercular cortex	Right	105	54	0	6	4.00	0.03 ± 0.28	−0.10 ± 0.20
***HED > non‐food***	No activated clusters after correction for multiple comparisons								
***LED > non‐food***									
Con baseline > Ex baseline	Frontal pole	Left	173	−36	48	10	4.56	0.07 ± 0.16	−0.11 ± 0.25
	Central opercular cortex	Right	141	56	0	8	4.05	0.14 ± 0.36	−0.12 ± 0.20
***HED > LED***									
Ex baseline > Con baseline	Frontal pole	Right	118	52	36	18	4.37	−0.09 ± 0.40	0.14 ± 0.37
***LED > HED***									
Con baseline > Ex baseline	Frontal pole	Right	118	52	36	18	4.37	0.09 ± 0.40	−0.14 ± 0.37

*Note*: Whole‐brain group‐level statistical analysis performed using a higher‐level fixed effects model in FMRIB's Expert Analysis Tool (FEAT) which included the corresponding cerebral blood flow map as a voxelwise (confound) explanatory variable (*n* = 23 participants). The *z*‐statistic image for each contrast was thresholded at *z* > 3.1 using a corrected cluster significance threshold of *p* < .05. Results represent brain region identified from Harvard–Oxford cortical or subcortical probabilistic atlases, right or left brain hemisphere, the number of voxels in each cluster (2.2 mm^3^; minimum cluster size of 10 voxels), and the coordinates in MNI space, *z* value and BOLD signal change (mean ± SD) for the peak statistical voxel.

Abbreviations: BOLD, blood‐oxygen‐level‐dependent; Con, control trial; Ex, exercise trial; HED, high‐ and very high‐energy density foods; LED, very low‐ and low‐energy density foods; MNI, Montreal Neurological Institute.

#### 3.4.2 CBF‐adjusted primary analysis (exercise post–pre versus control post–pre)

##### Whole‐brain analysis

**FIGURE 5:**
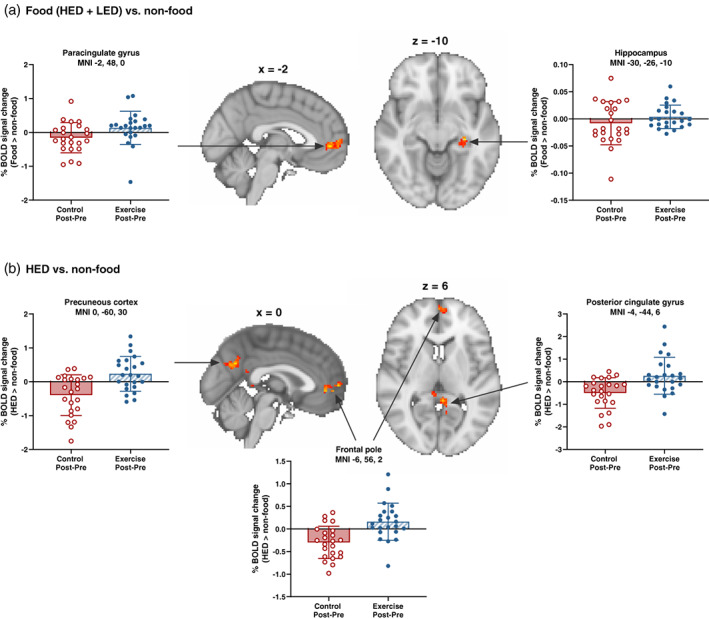
Voxel clusters showing greater blood‐oxygen‐level‐dependent (BOLD) activity in the pre‐to‐post change between the exercise versus control trial during a food cue reactivity task for the contrasts (a) food (HED + LED) versus non‐food cues and (b) HED versus non‐food cues (*n* = 23). The hippocampus cluster (a) was identified in the regions of interest analysis performed in Randomise (threshold‐free cluster enhancement, family‐wise corrected *p* value of *p* < .05); all other regions were detected in the whole brain analysis performed in FMRIBs Expert Analysis Tool (z > 3.1, corrected cluster significance threshold of *p* < .05). Brain maps presented in radiological convention with the right hemisphere shown on the left. Figures adjacent and underneath brain maps show the extracted BOLD signal change in the exercise (post–pre) and control (post–pre) trial for each identified cluster. Bars and error bars represent the mean ± SD and circles show the individual participant data values. Brain co‐ordinates are presented in MNI space for the peak statistical voxel. HED, high‐ and very high‐energy density foods; LED, very low‐ and low‐energy density foods; MNI, Montreal Neurological Institute.

Whole‐brain group analysis revealed exercise increased the neural response to food (HED + LED) versus non‐food cues and LED versus non‐food cues in the left paracingulate gyrus compared to control (Figure 5; Table 3). Greater pre‐to‐post reactivity to HED versus non‐food cues was identified in the left precuneous cortex, left frontal pole and left posterior cingulate gyrus in the exercise than control trial (Figure 5; Table 3). Pre‐to‐post activation in the left precentral gyrus was lower in response to HED versus LED cues and higher in response to LED versus HED cues in the exercise compared to control trial (Table 3).

**TABLE 3:** CBF‐adjusted whole‐brain analysis results of voxel clusters activated in the pre‐to‐post change between the exercise and control trials in each contrast (*CBF‐adjusted primary analysis*).

Contrast	Brain region	Hemisphere	No. of voxels	MNI brain coordinates			*z* value	BOLD signal change (%)	
				x	y	z		Control (post–pre)	Exercise (post–pre)
***Food (HED + LED) > non‐food***									
Ex post–pre > Con post–pre	Paracingulate gyrus	Left	226	−2	48	0	4.44	−0.16 ± 0.45	0.13 ± 0.49
***HED > non‐food***									
Ex post–pre > Con post–pre	Precuneous cortex	Left	225	0	−60	30	4.58	−0.40 ± 0.60	0.23 ± 0.51
	Frontal pole	Left	165	−6	56	2	4.39	−0.30 ± 0.36	0.16 ± 0.41
	Posterior cingulate gyrus	Left	116	−4	−44	6	4.32	−0.50 ± 0.68	0.26 ± 0.82
***LED > non‐food***									
Ex post–pre > Con post–pre	Paracingulate gyrus	Left	100	−10	52	2	4.17	−0.14 ± 0.33	0.13 ± 0.33
***HED > LED***									
Con post–pre > Ex post–pre	Precentral gyrus	Left	126	−60	0	32	4.28	0.11 ± 0.30	−0.24 ± 0.33
***LED > HED***									
Ex post–pre > Con post–pre	Precentral gyrus	Left	123	−60	2	32	4.24	−0.11 ± 0.28	0.22 ± 0.25

*Note*: Whole‐brain group‐level statistical analysis performed using a higher‐level mixed effects (FLAME 1 + 2) model in FMRIB's Expert Analysis Tool (FEAT) which included the corresponding cerebral blood flow map as a voxelwise (confound) explanatory variable (*n* = 23 participants). The *z*‐statistic image for each contrast was thresholded at *z* > 3.1 using a corrected cluster significance threshold of *p* < .05. Results represent brain region identified from Harvard–Oxford cortical or subcortical probabilistic atlases, right or left brain hemisphere, the number of voxels in each cluster (2.2 mm^3^; minimum cluster size of 10 voxels), and the coordinates in MNI space, z value and BOLD signal change (mean ± SD) for the peak statistical voxel.

Abbreviations: BOLD, blood‐oxygen‐level‐dependent; Con, control trial; Ex, exercise trial; HED, high‐ and very high‐energy density foods; LED, very low‐ and low‐energy density foods; MNI, Montreal Neurological Institute; post, fMRI scan performed after the exercise/rest period; pre, fMRI scan performed at baseline before the exercise/rest period.

##### Regions of interest analysis

Greater pre‐to‐post reactivity to food (HED + LED) versus non‐food cues was identified in the left hippocampus in the exercise compared to control trial (*z* score = 4.78; MNI coordinates: x = −30, y = −26, z = −10; size = 33 voxels; *p* = .035) (Figure 5). Between‐trial differences in food cue reactivity were not identified in any of the other a priori ROI masks (amygdala, hypothalamus, insula, nucleus accumbens, OFC and striatum).

### 3.5 Unadjusted BOLD response for food cue task

Analysis of food cue reactivity at baseline and in response to exercise without adjustment for CBF yielded broadly comparable findings to the CBF‐adjusted models and is presented in the supplementary results (Sections 2.2.2 and 2.2.3).

### 3.6 Exploratory correlations

**FIGURE 6:**
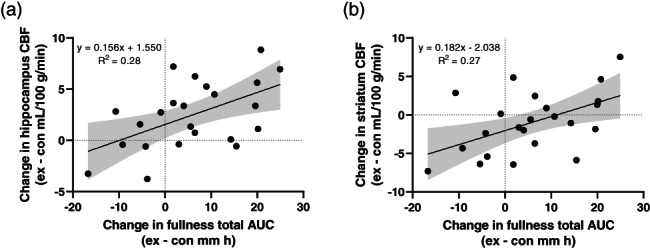
Correlation between the exercise‐induced change in (a) hippocampus and (b) striatum cerebral blood flow (CBF) measured using pseudo‐continuous arterial spin labelling (pCASL) and the exercise‐induced change in fullness time‐averaged total area under the curve (AUC) in 23 healthy men. Grey shaded area represents the 95% confidence interval of the regression line (black solid line).

The exercise‐induced change in time‐averaged total AUC for fullness was positively correlated with the between‐trial difference in hippocampus (*r* = 0.53 [95% CI 0.15, 0.77], *p* = .009) and striatum (*r* = 0.52 [95% CI 0.13, 0.77], *p* = .012) CBF (Figure 6). No other statistically significant correlations between net exercise energy expenditure or appetite perceptions and CBF (*r* = −0.38 to 0.41, *p* ≥ .055) or BOLD signal changes (*r* = −0.40 to 0.30, *p* ≥ .055) were identified.

## 4 DISCUSSION

The main findings from this study are that overall between‐trial differences in grey matter and regional CBF were apparent but the time‐course of CBF was not influenced directly by exercise in healthy young men. A single bout of running suppressed subjective appetite ratings and increased food cue reactivity in brain regions linked to attention, anticipation and encoding of reward, and episodic memory retrieval. However, the sensitivity of the exercise‐induced BOLD signal changes during a food cue reactivity task was not altered substantially after accounting for the underlying CBF.

The main effect of trial analysis revealed that CBF was higher in grey matter, the posterior insula and in the region of the amygdala/hippocampus, and lower in the medial OFC and dorsal striatum in the exercise versus control trial. This extends previous findings demonstrating pre‐to‐post exercise alterations in ASL derived CBF quantified globally and in regions linked to cognitive processing (MacIntosh et al., 2014; Mast et al., 2022; Olivo et al., 2021; Smith et al., 2010; Steventon et al., 2020) to show exercise‐related differences are apparent in key regions associated with food cue reactivity. Although CBF is a primary determinant of the BOLD phenomenon (Buxton et al., 2004), any residual effects of CBF fluctuations induced by exercise (Ogoh & Ainslie, 2009) could influence the BOLD response during proximal task‐based paradigms. Covarying out resting CBF has been shown to improve detection of the BOLD signal during a language task (Krishnamurthy et al., 2020); however, our findings show that the BOLD signal changes during the food cue reactivity task between trials were largely similar in models with and without adjustment for underlying CBF. Nevertheless, acquiring concurrent BOLD and CBF data may still be prudent to account for any subtle variability in CBF between scan sessions and/or participants that could confound the interpretation of exercise‐related brain food cue reactivity.

Despite identifying overall between‐trial differences in CBF, we did not detect any temporal patterns in grey matter or regional CBF when tracked ~8–28 min after exercise/rest supported by the absence of any trial‐by‐time interactions. This appears to contrast previous findings that have reported pre‐to‐post exercise changes in CBF when assessed at multiple time‐points after exercise. Specifically, greater hippocampus CBF has been reported 15‐, 40‐ and 60‐min after a moderate‐intensity exercise bout (Steventon et al., 2020), whereas lower grey matter, hippocampus and insula CBF has also been observed 10 min after exercise with the lower hippocampus CBF persisting at 40 min post‐exercise (MacIntosh et al., 2014). The inconsistency in the presence and direction of exercise‐induced CBF responses may partly reflect study design differences including scanning protocols, CBF assessment timing, exercise stimulus and participant characteristics. Previous studies are also limited by the absence of a control trial which our data suggests may be important given the time‐course of grey matter and regional CBF was largely similar between experimental trials. Nevertheless, the lack of any apparent time‐dependent changes in CBF after exercise in this study suggests food cue‐related BOLD acquisitions may not be time sensitive immediately after exercise.

Analysis of the BOLD data identified greater exercise‐induced food cue reactivity in the medial frontal pole, whereas reactivity to HED versus non‐food cues was lower in the middle frontal gyrus after exercise. Although knowledge of the precise function of these frontal cortical regions in human behaviour is still evolving, the medial frontal pole appears to hold distinct functions in self‐monitoring of emotions and external stimulus‐orientated thought (Bludau et al., 2014; Gilbert et al., 2005), whereas the middle frontal gyrus has been linked to inhibition in response to food stimuli (Nakata et al., 2008) and stimulus‐driven attention (Corbetta & Shulman, 2002). It is possible, therefore, that exercise may promote positive self‐reflections and responses to familiar foods whilst attenuating inhibition towards high value food stimuli. Whilst these regions have not been detected in previous acute exercise studies (Crabtree et al., 2014; Evero et al., 2012; Masterson et al., 2018; Saanijoki et al., 2018), lower OFC food cue reactivity has been reported after exercise (Crabtree et al., 2014). This prefrontal cortex region is a well‐established site for reward processing of food and food cues (Kringelbach, 2005) and is thought to interact with the neighbouring frontal pole (Koechlin & Hyafil, 2007).

The CBF‐adjusted primary model identified greater food cue reactivity in the paracingulate gyrus and posterior cingulate gyrus, located on the superior aspect and in the caudal region of the cingulate cortex, respectively, in addition to the precuneous cortex located adjacent to the posterior cingulate gyrus. Previous work has reported lower food cue reactivity in the posterior cingulate and cingulate gyrus after exercise (Crabtree et al., 2014), whereas greater exercise‐induced BOLD signal changes have been detected in the precuneus after exercise (Evero et al., 2012; Janse Van Rensburg et al., 2009). Our data support the latter finding, but the conflicting direction of BOLD signal change in cingulate cortex regions is ambiguous particularly considering our secondary sub‐analysis that provides an equivalent analysis to previous literature did not alter interpretation of the CBF‐adjusted primary model. Key functions of the precuneous are visuo‐spatial processing, attention shifts between objects, and episodic memory retrieval (Cavanna & Trimble, 2006). The latter function is shared by the posterior cingulate gyrus which also displays prominent connections to other brain regions implicated in attention (e.g., precuneus) and learning and motivation (e.g., anterior cingulate cortex and OFC) (Leech & Sharp, 2014). Consequently, the greater food cue reactivity observed in this study may reflect increased attention to food stimuli and anticipation of reward.

Alongside the elevated hippocampus CBF in the exercise trial, exercise evoked greater food cue responsiveness in this region of the temporal lobe which persisted after adjustment for CBF. This supports previous findings in children (Masterson et al., 2018) but is not reported universally with evidence of reduced hippocampus food cue reactivity observed after exercise in men (Crabtree et al., 2014). The hippocampus plays a critical role in integrating episodic meal‐related memories with information from internal signals (e.g., hunger, satiety) and external food cues to influence appetite and eating behaviour (Kanoski & Grill, 2017; Parent et al., 2022). Notably, episodic retrieval of meal‐related memories inhibits subsequent food intake (Parent et al., 2022), and greater hippocampus food cue reactivity has been reported in the post‐consumptive, but not the fasted state (Jones et al., 2021). Although participants were fasted throughout the visits in this study, the greater exercise‐induced hippocampus food cue reactivity occurred when subjective appetite was suppressed and, therefore, could reflect a response that might be expected to delay meal initiation. While this speculation requires confirmation as energy intake was not assessed, we observed a positive correlation between hippocampus CBF and perceptions of fullness and acute exercise has been shown previously to increase feeding latency despite not altering the total amount of energy consumed in the hours after exercise (King et al., 2013).

Our BOLD analysis also revealed lower reactivity to HED versus LED and greater reactivity to LED versus HED food images in response to exercise in the precentral gyrus. This region is primarily involved in motor responses but appears sensitive to food cue exposure (Huerta et al., 2014) and has been linked to the anticipation of and motor planning for food intake (Geliebter et al., 2006). A possible interpretation of our data is that exercise may elicit anticipation and motor planning about consuming foods of low over high energy value. The notion that exercise may preferentially increase and decrease reward‐related activation to low and high value foods, respectively, has been supported previously (Crabtree et al., 2014). Apart from the hippocampus cluster, we identified no activated clusters in other central reward‐related brain regions including the amygdala, insula, OFC and striatum which appears to contrast previous investigations when assessed within ~10 min of exercise completion (Crabtree et al., 2014; Evero et al., 2012). Our post‐condition scan was performed ~28–41 min after exercise/rest when appetite perceptions were returning towards control values and no acute exercise effect on food cue reactivity was reported previously when captured 80–110 min after exercise (Saanijoki et al., 2018). Thus, monitoring BOLD activity closer to the exercise bout may be required to detect robust responses in reward‐related brain regions.

Previous literature has demonstrated consistent but transient suppressions of appetite during and immediately after acute exercise bouts (Dorling et al., 2018). Our results corroborate these findings by showing suppressions in hunger and prospective food consumption, whereas fullness increased in response to exercise. In agreement with previous studies (Crabtree et al., 2014; Evero et al., 2012), no brain–behaviour relationships were identified between the exercise‐induced food cue reactivity and perceived appetite parameters. Although this suggests a potential disconnect between central brain and subjective appetite measures, the correlational analysis was exploratory and should be considered preliminary.

Key strengths of this study include the acquisition of adjacent pCASL and BOLD data and the food cue stimuli comprised distinct low and high hedonic value categories of known energy density which were confirmed to activate reward‐related brain regions. Furthermore, the pre‐intervention fMRI scan and the rest control trial allowed variability at baseline and without an exercise stimulus, respectively, to be captured appropriately in the analysis. A notable limitation is the recruitment of a small sample of healthy, lean young men and, therefore, future extension of this work to larger, more diverse and clinical populations is encouraged including women and in individuals with excess adiposity who typically display heightened food cue reactivity (Meng et al., 2020). Circulating appetite‐related hormone concentrations and ad libitum energy intake were not assessed in this study but future work integrating these measures alongside appetite‐related brain responses would provide a more holistic insight into exercise and appetite interactions. Apart from heart rate monitoring during exercise, physiological cardio‐pulmonary markers that influence CBF were not measured. Future studies should integrate regular physiological measurements such as arterial blood pressure, heart rate, respiration rate and oxygen saturation to improve the sensitivity to detect the cerebrovascular contribution to food cue‐induced brain responses to exercise. Finally, the masks for the ROI analysis were created using probabilistic brain atlases to better accommodate inter‐individual anatomical variability but may have reduced the ability to detect the precise location of activated voxel clusters close to the boundary of structures.

## 5 CONCLUSION

Despite identification of overall between‐trial differences in CBF, acute vigorous‐intensity running did not directly influence the time‐course of CBF suggesting food‐cue related BOLD acquisitions immediately after exercise may not be time sensitive at least in relation to any residual influence of CBF. Acute running transiently suppressed appetite and increased food cue reactivity in brain regions implicated in attention, anticipation and encoding of reward, and episodic memory retrieval independent of CBF. These data provide insights into the immediate effects of exercise on central appetite responses and highlight important methodological considerations for future work exploring the interaction of exercise with brain food‐cue responsiveness.

## AUTHOR CONTRIBUTIONS

**Alice E. Thackray:** conceptualisation, methodology, formal analysis, investigation, writing – original draft, writing – review and editing, visualisation, project administration. **Elanor C. Hinton:** conceptualisation, methodology, formal analysis, resources, writing – review and editing. **Turki M. Alanazi:** validation, investigation, writing – review and editing, funding acquisition. **Abdulrahman M. Dera:** validation, investigation, writing – review and editing, funding acquisition. **Kyoko Fujihara:** methodology, writing – review and editing. **Julian P. Hamilton‐Shield:** writing – review and editing, supervision. **James A. King:** writing – review and editing. **Fiona E. Lithander:** methodology, writing – review and editing. **Masashi Miyashita:** writing – review and editing, supervision. **Julie Thompson:** methodology, investigation, writing – review and editing. **Paul S. Morgan:** methodology, resources, writing – review and editing, supervision. **Melanie J. Davies:** writing – review and editing, funding acquisition. **David J. Stensel:** conceptualisation, methodology, resources, writing – review and editing, supervision, funding acquisition.

## CONFLICT OF INTEREST STATEMENT

The authors declare no conflicts of interest.

## Supporting information


**Data S1:** Supporting Information

## Data Availability

The de‐identified neuroimaging, physiological and behavioural data generated in this study are available from the corresponding author upon reasonable request.
