# Supplementary material for: Exploring the acute effects of running on cerebral blood flow and food cue reactivity in healthy young men using functional magnetic resonance imaging
Source: Hum Brain Mapp. 2023 May 5;44(9):3815–32. doi: 10.1002/hbm.26314 (PMC10203797; doi:10.1002/hbm.26314)
Supplement: Supplementary file 1 — Data S1: Supporting Information [file HBM-44-3815-s001.docx]

# Supplementary material

**Title: Exploring the acute effects of running on cerebral blood flow and food cue reactivity in healthy young men using functional magnetic resonance imaging**

**Authors:** Alice E. Thackray, Elanor C. Hinton, Turki M. Alanazi, Abdulrahman M. Dera, Kyoko Fujihara, Julian P. Hamilton-Shield, James A. King, Fiona E. Lithander, Masashi Miyashita, Julie Thompson, Paul S. Morgan, Melanie J. Davies, David J. Stensel

# Supplementary methods

- 1. ***Pre-processing stages***

All DICOM images were exported from the MRI scanner and converted to NIFTI format using the contemporary version of dcm2niix (Li et al. 2016). Non-brain structures were removed from the anatomical T1-weighted images using fsl_anat. Registration of functional data to anatomical and standard space (MNI152_T1_2mm_brain) images was performed using FMRIB’s Linear (FLIRT; Jenkinson et al. 2002) and Non-linear (FNIRT; Andersson et al. 2007) Image Registration Tools. Non-brain tissue was removed from the cerebral blood flow (CBF) maps generated from the pseudo-continuous arterial spin labelling (pCASL) scans using BET (Smith 2002). A grey matter mask to use in the pCASL analysis was generated by segmenting grey matter tissue from the anatomical T1-weighted image using FMRIB's Automated Segmentation Tool (FAST) (Zhang et al. 2001).

Additional pre-processing of task-based functional data was conducted using FMRIBs Expert Analysis Tool (FEAT) (Woolrich et al. 2001, 2004). Pre-processing steps applied involved motion correction using MCFLIRT (Jenkinson et al. 2002), spatial smoothing using a Gaussian kernel with full width half maximum of 5 mm, global intensity normalisation, susceptibility-induced distortion correction using topup (Andersson et al. 2003; Smith et al. 2004), and high-pass filtering using a cut-off of 90 s. Task-based functional data were also denoised to remove motion-related artefacts using ICA-AROMA (Pruim et al. 2015).

**Supplementary Table 1.** Images presented during the food cue reactivity paradigm.

| **Very low- and low-energy density foods (LED)** | |  | **High- and very high-energy**  **density foods (HED)** | |  | **Non-food objects** | |
| --- | --- | --- | --- | --- | --- | --- | --- |
| **Image number** | **Description** |  | **Image number** | **Description** |  | **Image number** | **Description** |
| 192 | Apple |  | 1 | Cheesecake |  | 1008 | Bucket |
| 199 | Watermelon |  | 4 | Cookie |  | 1012 | Cushion |
| 202 | Blueberries |  | 16 | Pancakes |  | 1014 | Ladder |
| 221 | Orange |  | 41 | Donut |  | 1015 | Hole punch |
| 222 | Strawberries |  | 107 | Chocolate cake |  | 1017 | Note pad |
| 254 | Figs |  | 116 | Ice cream |  | 1018 | Paint brushes |
| 255 | Pomegranate |  | 123 | Lollipops |  | 1019 | Drawing pins |
| 256 | Grapefruits |  | 137 | Muffin |  | 1024 | Screwdriver |
| 280 | Cherries |  | 148 | Shortbread |  | 1028 | Stapler |
| 281 | Green grapes |  | 151 | Lemon cake |  | 1031 | Telephone |
| 282 | Banana |  | 153 | Cola bottles |  | 1034 | Leaf |
| 284 | Red grapes |  | 159 | Chocolate cake bar |  | 1038 | Hairbrush |
| 393 | Honeydew melon |  | 180 | Muesli bar |  | 1046 | Brick |
| 402 | Pear |  | 286 | Chocolate |  | 1050 | Chess pieces |
| 407 | Blackberries |  | 287 | Chocolate bar |  | 1094 | Pencil case |
| 413 | Kiwis |  | 295 | Toblerone |  | 1096 | Car rim |
| 453 | Peach |  | 296 | Chocolate sweets |  | 1130 | Screw and nuts |
| 478 | Pineapple |  | 344 | Brownie |  | 1140 | Calculator |
| 479 | Papaya |  | 507 | Waffle |  | 1144 | Book |
| 531 | Raspberries |  | 511 | Chocolate teacake |  | 1146 | Clipboard |
| 195 | Cucumber |  | 26 | Crisps |  | 1147 | Briefcase |
| 233 | Tomatoes |  | 64 | Butter |  | 1149 | Magnifying glass |
| 250 | Broccoli |  | 110 | Cashew nuts |  | 1155 | Paper clips |
| 252 | Lettuce |  | 117 | Crisps |  | 1187 | Snail shell |
| 258 | Radishes |  | 155 | Crackers |  | 1208 | Broom |
| 262 | Celery |  | 175 | Brie |  | 1218 | Chair |
| 264 | Mushrooms |  | 176 | Salami |  | 1240 | Umbrella |
| 265 | Zucchini |  | 183 | Peanuts |  | 1250 | Thread |
| 270 | Sweetcorn |  | 189 | Bread with chocolate spread |  | 1256 | Torch |
| 301 | Roasted chicken |  | 193 | Rye crisp bread |  | 1267 | Light bulb |
| 317 | Roasted potatoes |  | 236 | Rice |  | 1273 | Cabinet |
| 334 | Carrots |  | 244 | Rice cakes |  | 1274 | Watering can |
| 359 | Asparagus |  | 294 | Popcorn |  | 1277 | Candle |
| 418 | Cauliflower |  | 351 | Croissant |  | 1282 | Leaf |
| 424 | Peas |  | 372 | Cereals |  | 1284 | Flower |
| 434 | Leek |  | 450 | Hazelnuts |  | 1293 | Pine-cone |
| 435 | Brussel sprouts |  | 494 | Pretzel |  | 1301 | Flower |
| 442 | Bell pepper |  | 515 | Emmental |  | 1309 | Twig |
| 455 | Spinach |  | 539 | Almonds |  | 1313 | Bicycle |
| 564 | Sushi |  | 547 | Hot dog |  | 1314 | Hot-air balloon |

Images were obtained from a freely available database (Blechert et al. 2014, 2019).

**Supplementary Table 2.** Nutritional information and image characteristics of the food and non-food images presented during the food cue reactivity paradigm.

|  | **Very low- and low-energy density foods (LED)** | **High- and very high-energy density foods (HED)** | **Non-food objects** |
| --- | --- | --- | --- |
| ***Nutritional information*** |  |  |  |
| Portion (g) | 160 (91–321) | 62 (35–103) |  |
| Energy (kcal) | 73 (30–112) | 265 (169–462) |  |
| Energy density (kcal/g) | 0.35 (0.17–0.57) | 4.45 (3.80–5.26) |  |
| Protein (g) | 2.00 (0.71–5.36) | 3.76 (1.93–8.21) |  |
| Carbohydrate (g) | 11.34 (3.75–18.44) | 22.17 (11.60–42.08) |  |
| Fat (g) | 0.45 (0.26–0.89) | 12.48 (4.75–25.95) |  |
| ***Image characteristics*** |  |  |  |
| Red colour | 0.44 (0.37–0.53) | 0.47 (0.42–0.52) | 0.43 (0.35–0.47) |
| Green colour | 0.35 (0.29–0.39) | 0.33 (0.30–0.35) | 0.33 (0.31–0.36) |
| Blue colour | 0.19 (0.15–0.23) | 0.20 (0.16–0.24) | 0.25 (0.19–0.31) |
| Object size | 0.28 (0.21–0.37) | 0.30 (0.24–0.38) | 0.29 (0.23–0.34) |
| Brightness | 29.6 (21.0–44.9) | 30.4 (22.7–38.9) | 30.9 (23.1–42.8) |
| Contrast | 52.8 (39.8–58.7) | 51.7 (39.1–60.7) | 54.7 (40.0–64.8) |
| Normalised complexity | 0.25 (0.20–0.34) | 0.26 (0.20–0.34) | 0.21 (0.16–0.33) |

Values are median (interquartile range). A threshold of ≤ 1.5 kcal/g and ≥ 2.26 kcal/g was applied to classify LED and HED foods, respectively (Vernarelli et al. 2018). Nutritional information (LED and HED only) and the characteristics of each image were extracted from the information accompanying the image database (Blechert et al. 2014, 2019).

Colour represents the proportional contribution of each colour averaged across non-white pixels. Object size represents the proportion of non-white pixels to the total number of pixels. Brightness represents the difference between the mean luminance of non-white pixels (after converting to grey scale) versus the white background with higher values indicative of more salient objects. Contrast represents the standard deviation of luminance across non-white pixels (after converting to grey scale) with higher values indicative of a greater range of light-to-dark pixels. Normalised complexity represents the proportion of outline-related pixels in the image divided by the number of non-white pixels.

1. **Supplementary results**
   1. ***Post-study survey responses***

High levels of familiarity were evident for the 120 images presented during the food cue reactivity task with participants indicating familiarity with 96% of the images (category breakdown: LED 96%; HED 96%; non-food objects 97%). An average of 79% of the food items presented during the food cue reactivity task had been consumed at least once by participants in the previous 12 months (category breakdown: LED 82%; HED 76%) (Supplementary Figure 1A). On average, 69% of the food items were rated with a likeness score between 6 and 10 (category breakdown: LED 66%; HED 73%) (Supplementary Figure 1B).


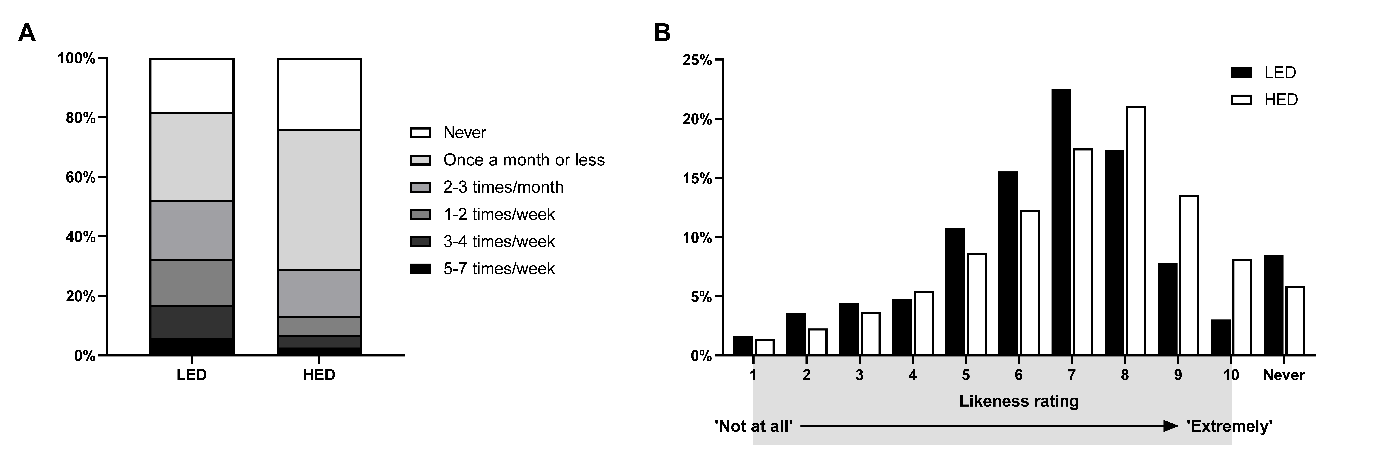


**Supplementary Figure 1.** Summary results of the post-study survey completed by 23 men showing the (A) frequency of consumption and (B) likeness rating for the food images presented during the food cue reactivity task. Values represent the percentage of food images selected in each category. Participants were asked to respond to the questions: (A) ‘Over the past 12 months, how often did you consume this food?’ and (B) ‘How much do you like the taste of this food?’. LED, very low- and low-energy density foods; HED, high- and very high-energy density foods.

- 1. ***BOLD response for food cue task unadjusted for CBF***
     1. *Preliminary baseline analysis: first food cue exposure*

*Whole brain analysis.* Greater reactivity to food (HED+LED) vs. non-food cues was identified in the left posterior middle temporal gyrus, right superior frontal gyrus and left posterior supramarginal gyrus (Supplementary Table 3). Greater activation was found in the left superior lateral occipital cortex and left frontal pole in response to HED vs. non-food cues, and in the left posterior middle temporal gyrus, left superior lateral occipital cortex and left anterior insular cortex in response to LED vs. non-food cues (Supplementary Table 3). Greater bilateral activation was also detected in the inferior lateral occipital cortex in response to HED vs. LED cues (Supplementary Table 3).

*Regions of interest (ROI) analysis.* Greater activation in the anterior insular cortex was detected in response to food (HED+LED) vs. non-food cues (P = 0.026, uncorrected for multiple comparisons) and LED vs. non-food cues (P = 0.019, uncorrected for multiple comparisons) (Supplementary Table 3). Furthermore, greater reactivity to LED cues was detected in the left dorsal striatum compared to both non-food cues (caudate nucleus; P = 0.011, uncorrected for multiple comparisons) and HED cues (putamen; P = 0.009, uncorrected for multiple comparisons) (Supplementary Table 3). No other clusters surpassed the threshold of 10 continuous voxels in the *a priori* ROIs.

- - 1. *Comparative baseline analysis (whole brain only)*

*Whole-brain analysis.* Activation in the frontal pole was lower at baseline in the exercise than control trial in response to food (HED+LED) vs. non-food cues, LED vs. non-food cues, and LED vs. HED cues, but was higher in the exercise than control trial in response to HED vs. LED cues (Supplementary Table 4). In the exercise compared to control trial, activation in the right central opercular cortex was lower at baseline in response to food (HED+LED) vs. non-food cues and LED vs. non-food cues, and lower reactivity to HED vs. non-food cues was identified in the left posterior cingulate gyrus (Supplementary Table 4).

- - 1. *Primary analysis (exercise post-pre vs. control post-pre)*

*Whole brain analysis.* Whole-brain group analysis revealed exercise increased the neural response to food (HED+LED) vs. non-food cues in the left paracingulate gyrus compared to control (Supplementary Table 5). This region also showed greater pre-to-post reactivity to HED vs. non-food cues and LED vs. non-food cues in the exercise than control trial (Supplementary Table 5). In the exercise compared to control trial, pre-to-post activation in the left precuneous cortex and left posterior cingulate gyrus was higher in response to HED vs. non-food cues, and activation was higher in the left inferior frontal gyrus (pars triangularis) in response to LED vs. non-food cues (Supplementary Table 5). Pre-to-post activation in the left precentral gyrus was lower in response to HED vs. LED cues and higher in response to LED vs. HED cues in the exercise compared to control trial (Supplementary Table 5).

*Regions of interest (ROI) analysis.* Greater pre-to-post reactivity to food (HED+LED) vs. non-food cues was identified in the left hippocampus in the exercise compared to control trial (P = 0.029, corrected for multiple comparisons) (Supplementary Table 5). Between-trial differences in food cue reactivity were not identified in any of the other *a priori* ROI masks (amygdala, hypothalamus, insula, nucleus accumbens, OFC and striatum).

- - 1. *Secondary sub-analysis (exercise post vs. control post; whole brain only)*

*Whole brain analysis.* Greater reactivity to food cues after exercise compared to after control was observed in the left frontal pole (food [HED+LED] vs. non-food cues, HED vs. non-food cues), left posterior cingulate gyrus (food [HED+LED] vs. non-food cues) and right precuneous cortex (HED vs. non-food cues) (Supplementary Table 6). After exercise compared to after control, activation in the right superior lateral occipital cortex was lower in response to food (HED+LED) vs. non-food cues and HED vs. non-food cues (Supplementary Table 6). Additional clusters that were lower after exercise than after control were also identified in the right middle frontal gyrus and right angular gyrus in response to HED vs. non-food cues (Supplementary Table 6). Activation in the left precentral gyrus and right postcentral gyrus was lower after exercise than after control in response to HED vs. LED cues (Supplementary Table 6). Both these regions showed greater reactivity to LED vs. HED cues after exercise than after control (Supplementary Table 6).

**Supplementary Table 3.** Whole-brain and regions of interest analysis results of voxel clusters activated in the first exposure to the food cue reactivity task (*preliminary baseline analysis)*.

| **Contrast** | **Brain region** | **Hemisphere** | **No. of voxels** | **MNI brain coordinates** | | | ***z* value** | **BOLD signal change (%)** |
| --- | --- | --- | --- | --- | --- | --- | --- | --- |
|  |  |  |  | **x** | **y** | **z** |  |  |
| ***Food (HED + LED) > non-food*** | Posterior middle temporal gyrus | Left | 190 | -66 | -24 | -4 | 4.88 | 0.22 ± 0.44 |
|  | Superior frontal gyrus | Right | 147 | 2 | 46 | 34 | 4.57 | 0.09 ± 0.30 |
|  | Posterior supramarginal gyrus | Left | 113 | -62 | -52 | 32 | 4.75 | 0.13 ± 0.31 |
|  | Insular cortex (anterior)* | Left | 26 | -38 | 6 | -12 | 4.62 | 0.14 ± 0.14 |
| ***HED > non-food*** | Superior lateral occipital cortex | Left | 181 | -38 | -84 | 12 | 5.32 | 0.20 ± 0.29 |
|  | Frontal pole | Left | 114 | -56 | 36 | 10 | 4.11 | 0.26 ± 0.56 |
| ***LED > non-food*** | Posterior middle temporal gyrus | Left | 272 | -64 | -24 | -6 | 4.59 | 0.21 ± 0.30 |
|  | Superior lateral occipital cortex | Left | 127 | -52 | -60 | 32 | 4.39 | 0.21 ± 0.24 |
|  | Insular cortex (anterior) | Left | 89 | -38 | 4 | -12 | 4.42 | 0.18 ± 0.21 |
|  | Insular cortex (anterior)* | Left | 50 | -40 | 4 | -12 | 4.58 | 0.20 ± 0.22 |
|  | Insular cortex (anterior)* | Right | 27 | 46 | 12 | -10 | 4.50 | 0.15 ± 0.18 |
|  | Striatum (dorsal, caudate nucleus)* | Left | 19 | -8 | 10 | -2 | 4.91 | 0.24 ± 0.24 |
| ***HED > LED*** | Inferior lateral occipital cortex | Right | 462 | 30 | -84 | 6 | 5.44 | 0.09 ± 0.19 |
|  | Inferior lateral occipital cortex | Left | 198 | -32 | -86 | 6 | 5.15 | 0.16 ± 0.18 |
| ***LED > HED*** | No activated clusters in whole brain analysis after correction for multiple comparisons | | | | | | | |
|  | Striatum (dorsal, putamen)* | Left | 18 | -16 | 6 | -12 | 5.11 | 0.14 ± 0.17 |

The first exposure to the food cue reactivity task was in the baseline (pre) scan of the control (*n* = 12) or exercise (*n* = 11) trial.

* Denotes clusters detected in the regions of interest analysis; all other clusters identified in the whole-brain analysis (*n* = 23 participants).

Whole-brain group-level statistical analysis performed using a higher-level fixed effects model in FMRIB’s Expert Analysis Tool (FEAT). The *z*-statistic image for each contrast was thresholded at *z* > 3.1 using a corrected cluster significance threshold of P < 0.05. Regions of interest (ROI) analysis performed using a non-parametric permutation approach in Randomise applying threshold-free cluster enhancement (TFCE) and a family-wise error corrected P value of P < 0.05. Due to the exploratory nature of the analysis, no Bonferroni correction was applied for multiple ROI comparisons.

Results represent brain region identified from Harvard-Oxford cortical or subcortical probabilistic atlases, right or left brain hemisphere, the number of voxels in each cluster (2.2 mm^3^; minimum cluster size of 10 voxels), and the coordinates in MNI space, *z* value and BOLD signal change (mean ± SD) for the peak statistical voxel.

MNI, Montreal Neurological Institute; BOLD, blood-oxygen-level-dependent; HED, high- and very high-energy density foods; LED, very low- and low-energy density foods.

**Supplementary Table 4.** Whole-brain analysis results of voxel clusters activated in the baseline difference between the exercise and control trials in each contrast (*unadjusted comparative baseline analysis)*.

| **Contrast** | **Brain region** | **Hemisphere** | **No. of voxels** | **MNI brain coordinates** | | | ***z* value** | **BOLD signal change (%)** | |
| --- | --- | --- | --- | --- | --- | --- | --- | --- | --- |
|  |  |  |  | **x** | **y** | **z** |  | **Control (baseline)** | **Exercise (baseline)** |
| ***Food (HED + LED) > non-food*** |  |  |  |  |  |  |  |  |  |
| Con baseline > Ex baseline | Central opercular cortex | Right | 103 | 56 | 0 | 6 | 4.31 | 0.06 ± 0.32 | -0.12 ± 0.19 |
|  | Frontal pole | Left | 90 | -32 | 46 | 10 | 4.12 | 0.02 ± 0.16 | -0.09 ± 0.23 |
| ***HED > non-food*** |  |  |  |  |  |  |  |  |  |
| Con baseline > Ex baseline | Posterior cingulate gyrus | Left | 83 | -2 | -38 | 42 | 4.96 | 0.10 ± 0.18 | -0.06 ± 0.51 |
| ***LED > non-food*** |  |  |  |  |  |  |  |  |  |
| Con baseline > Ex baseline | Central opercular cortex | Right | 149 | 58 | 0 | 6 | 4.27 | 0.15 ± 0.47 | -0.13 ± 0.29 |
|  | Frontal pole | Left | 144 | -38 | 46 | 14 | 4.42 | 0.08 ± 0.26 | -0.18 ± 0.30 |
| ***HED > LED*** |  |  |  |  |  |  |  |  |  |
| Ex baseline > Con baseline | Frontal pole | Right | 126 | 50 | 36 | 16 | 4.73 | -0.10 ± 0.32 | 0.12 ± 0.33 |
| ***LED > HED*** |  |  |  |  |  |  |  |  |  |
| Con baseline > Ex baseline | Frontal pole | Right | 126 | 50 | 36 | 16 | 4.73 | 0.10 ± 0.32 | -0.12 ± 0.33 |

Whole-brain group-level statistical analysis performed using a higher-level fixed effects model in FMRIB’s Expert Analysis Tool (FEAT) with no adjustment for cerebral blood flow (*n* = 23 participants). The *z*-statistic image for each contrast was thresholded at *z* > 3.1 using a corrected cluster significance threshold of P < 0.05.

Results represent brain region identified from Harvard-Oxford cortical or subcortical probabilistic atlases, right or left brain hemisphere, the number of voxels in each cluster (2.2 mm^3^; minimum cluster size of 10 voxels), and the coordinates in MNI space, *z* value and BOLD signal change (mean ± SD) for the peak statistical voxel.

MNI, Montreal Neurological Institute; BOLD, blood-oxygen-level-dependent; HED, high- and very high-energy density foods; LED, very low- and low-energy density foods; Con, control trial; Ex, exercise trial.

**Supplementary Table 5.** Whole-brain and regions of interest analysis results of voxel clusters activated in the pre-to-post change between the exercise and control trials in each contrast (*unadjusted primary analysis*).

| **Contrast** | **Brain region** | **Hemisphere** | **No. of voxels** | **MNI brain coordinates** | | | ***z* value** | **BOLD signal change (%)** | |
| --- | --- | --- | --- | --- | --- | --- | --- | --- | --- |
|  |  |  |  | **x** | **y** | **z** |  | **Control**  **(post – pre)** | **Exercise**  **(post – pre)** |
| ***Food (HED + LED) > non-food*** |  |  |  |  |  |  |  |  |  |
| Ex post-pre > Con post-pre | Paracingulate gyrus | Left | 252 | -10 | 52 | 2 | 4.64 | -0.17 ± 0.30 | 0.12 ± 0.29 |
|  | Hippocampus* | Left | 34 | -30 | -26 | -10 | 4.91 | -0.009 ± 0.040 | 0.004 ± 0.022 |
| ***HED > non-food*** |  |  |  |  |  |  |  |  |  |
| Ex post-pre > Con post-pre | Precuneous cortex | Left | 307 | 0 | -62 | 28 | 4.56 | -0.35 ± 0.54 | 0.22 ± 0.51 |
|  | Paracingulate gyrus | Left | 261 | -8 | 54 | -2 | 4.70 | -0.20 ± 0.33 | 0.23 ± 0.42 |
|  | Posterior cingulate gyrus | Left | 136 | -4 | -42 | 6 | 4.21 | -0.39 ± 0.72 | 0.38 ± 0.81 |
| ***LED > non-food*** |  |  |  |  |  |  |  |  |  |
| Ex post-pre > Con post-pre | Paracingulate gyrus | Left | 127 | -8 | 54 | 0 | 4.36 | -0.15 ± 0.42 | 0.20 ± 0.45 |
|  | Inferior frontal gyrus, pars triangularis | Left | 78 | -40 | 34 | 10 | 4.36 | -0.22 ± 0.36 | 0.13 ± 0.35 |
| ***HED > LED*** |  |  |  |  |  |  |  |  |  |
| Con post-pre > Ex post-pre | Precentral gyrus | Left | 134 | -60 | -2 | 30 | 4.40 | 0.13 ± 0.40 | -0.29 ± 0.50 |
| ***LED > HED*** |  |  |  |  |  |  |  |  |  |
| Ex post-pre > Con post-pre | Precentral gyrus | Left | 129 | -60 | -4 | 30 | 4.32 | -0.15 ± 0.49 | 0.29 ± 0.46 |

* Denotes clusters detected in the regions of interest analysis; all other clusters identified in the whole-brain analysis (*n* = 23 participants).

Whole-brain group-level statistical analysis performed using a higher-level mixed effects (FLAME 1+2) model in FMRIB’s Expert Analysis Tool (FEAT) with no adjustment for cerebral blood flow. The *z*-statistic image for each contrast was thresholded at *z* > 3.1 using a corrected cluster significance threshold of P < 0.05. Regions of interest (ROI) analysis performed using a non-parametric permutation approach in Randomise applying threshold-free cluster enhancement (TFCE) and a family-wise error corrected P value of P < 0.05. A Bonferroni correction was applied to account for multiple ROI comparisons.

Results represent brain region identified from Harvard-Oxford cortical or subcortical probabilistic atlases, right or left brain hemisphere, the number of voxels in each cluster (2.2 mm^3^; minimum cluster size of 10 voxels), and the coordinates in MNI space, *z* value and BOLD signal change (mean ± SD) for the peak statistical voxel.

MNI, Montreal Neurological Institute; BOLD, blood-oxygen-level-dependent; post, fMRI scan performed after the exercise/rest period; pre, fMRI scan performed at baseline before the exercise/rest period; HED, high- and very high-energy density foods; LED, very low- and low-energy density foods; Ex, exercise trial; Con, control trial.

**Supplementary Table 6.** Whole-brain analysis results of voxel clusters activated in the difference between the exercise post and control post scans in each contrast (*unadjusted secondary sub-analysis*).

| **Contrast** | **Brain region** | **Hemisphere** | **No. of voxels** | **MNI brain coordinates** | | | ***z* value** | **BOLD signal change (%)** | |
| --- | --- | --- | --- | --- | --- | --- | --- | --- | --- |
|  |  |  |  | **x** | **y** | **z** |  | **Control (post)** | **Exercise (post)** |
| ***Food (HED + LED) > non-food*** |  |  |  |  |  |  |  |  |  |
| Ex post > Con post | Frontal pole | Left | 292 | -4 | 60 | 4 | 4.71 | -0.11 ± 0.26 | 0.11 ± 0.31 |
|  | Posterior cingulate gyrus | Left | 77 | -2 | -52 | 16 | 4.15 | -0.08 ± 0.27 | 0.19 ± 0.37 |
| Con post > Ex post | Superior lateral occipital cortex | Right | 103 | 36 | -62 | 48 | 4.44 | -0.003 ± 0.33 | -0.12 ± 0.37 |
| ***HED > non-food*** |  |  |  |  |  |  |  |  |  |
| Ex post > Con post | Frontal pole | Left | 389 | -2 | 60 | 4 | 5.02 | -0.14 ± 0.74 | 0.09 ± 0.42 |
|  | Precuneous cortex | Right | 149 | 2 | -60 | 28 | 4.31 | -0.19 ± 0.45 | 0.14 ± 0.40 |
| Con post > Ex post | Superior lateral occipital cortex | Right | 221 | 36 | -62 | 46 | 4.94 | 0.08 ± 0.25 | -0.12 ± 0.44 |
|  | Middle frontal gyrus | Right | 113 | 30 | 14 | 58 | 4.73 | 0.13 ± 0.22 | -0.13 ± 0.27 |
|  | Angular gyrus | Right | 83 | 60 | -52 | 40 | 3.97 | 0.06 ± 0.33 | -0.21 ± 0.39 |
| ***LED > non-food*** | No activated clusters after correction for multiple comparisons | | | | | | | | |
| ***HED > LED*** |  |  |  |  |  |  |  |  |  |
| Con post > Ex post | Precentral gyrus | Left | 181 | -62 | -2 | 24 | 4.54 | 0.08 ± 0.27 | -0.27 ± 0.36 |
|  | Postcentral gyrus | Right | 79 | 40 | -24 | 68 | 3.72 | 0.02 ± 0.67 | -0.37 ± 0.60 |
| ***LED > HED*** |  |  |  |  |  |  |  |  |  |
| Ex post > Con post | Precentral gyrus | Left | 181 | -62 | -2 | 24 | 4.54 | -0.08 ± 0.27 | 0.27 ± 0.36 |
|  | Postcentral gyrus | Right | 79 | 40 | -24 | 68 | 3.72 | -0.02 ± 0.67 | 0.37 ± 0.60 |

Whole-brain group-level statistical analysis performed using a higher-level fixed effects model in FMRIB’s Expert Analysis Tool (FEAT) with no adjustment for cerebral blood flow (*n* = 23 participants). The *z*-statistic image for each contrast was thresholded at *z* > 3.1 using a corrected cluster significance threshold of P < 0.05.

Results represent brain region identified from Harvard-Oxford cortical or subcortical probabilistic atlases, right or left brain hemisphere, the number of voxels in each cluster (2.2 mm^3^; minimum cluster size of 10 voxels), and the coordinates in MNI space, *z* value and BOLD signal change (mean ± SD) for the peak statistical voxel.

MNI, Montreal Neurological Institute; BOLD, blood-oxygen-level-dependent; post, fMRI scan performed after the exercise/rest periods; HED, high- and very high-energy density foods; LED, very low- and low-energy density foods; Ex, exercise trial; Con, control trial.

**References**

Andersson, J.L.R., Jenkinson, M., Smith, S., 2007. Non-linear registration, aka spatial normalisation. FMRIB technical report TR07JA2. Available at: <https://www.fmrib.ox.ac.uk/datasets/techrep/tr07ja2/tr07ja2.pdf>.

Andersson, J.L., Skare, S., Ashburner, J., 2003. How to correct susceptibility distortions in spin-echo echo-planar images: application to diffusion tensor imaging. NeuroImage. 20, 870–888. <https://doi.org/10.1016/S1053-8119(03)00336-7>.

Blechert, J., Lender, A., Polk, S., Busch, N. A., Ohla, K., 2019. Food-Pics_Extended-An image database for experimental research on eating and appetite: additional images, normative ratings and an updated review. Front. Psychol. 10, 307. <https://doi.org/10.3389/fpsyg.2019.00307>.

Blechert, J., Meule, A., Busch, N. A., Ohla, K., 2014. Food-pics: an image database for experimental research on eating and appetite. Front. Psychol. 5, 617. <https://doi.org/10.3389/fpsyg.2014.00617>.

Jenkinson, M., Bannister, P., Brady, M., Smith, S., 2002. Improved optimization for the robust and accurate linear registration and motion correction of brain images. NeuroImage. 17, 825–841. <https://doi.org/10.1016/s1053-8119(02)91132-8>.

Li, X., Morgan, P.S., Ashburner, J., Smith, J., Rorden, C., 2016. The first step for neuroimaging data analysis: DICOM to NIfTI conversion. J. Neurosci. Methods. 264, 47–56. <https://doi.org/10.1016/j.jneumeth.2016.03.001>.

Pruim, R., Mennes, M., van Rooij, D., Llera, A., Buitelaar, J.K., Beckmann, C.F., 2015. ICA-AROMA: a robust ICA-based strategy for removing motion artifacts from fMRI data. NeuroImage, 112, 267–277. <https://doi.org/10.1016/j.neuroimage.2015.02.064>.

Smith, S.M., 2002. Fast robust automated brain extraction. Hum. Brain. Mapp. 17, 143–155. <https://doi.org/10.1002/hbm.10062>.

Smith, S.M., Jenkinson, M., Woolrich, M.W., Beckmann, C.F., Behrens, T.E., Johansen-Berg, H., Bannister, P.R., De Luca, M., Drobnjak, I., Flitney, D.E., Niazy, R.K., Saunders, J., Vickers, J., Zhang, Y., De Stefano, N., Brady, J.M., Matthews, P.M., 2004. Advances in functional and structural MR image analysis and implementation as FSL. NeuroImage. 23, S208–S219. <https://doi.org/10.1016/j.neuroimage.2004.07.051>.

Vernarelli, J.A., Mitchell, D.C., Rolls, B.J., Hartman, T.J., 2018. Dietary energy density and obesity: how consumption patterns differ by body weight status. Eur. J. Nutr. 57, 351–361. <https://doi.org/10.1007/s00394-016-1324-8>.

Woolrich, M.W., Behrens, T.E.J., Beckmann, C.F., Jenkinson, M., Smith, S.M., 2004. Multilevel linear modelling for FMRI group analysis using Bayesian inference. NeuroImage, 21, 1732–1747. <http://doi.org/10.1016/j.neuroimage.2003.12.023>.

Woolrich, M.W., Ripley, B.D., Brady, M., Smith, S.M., 2001. Temporal autocorrelation in univariate linear modeling of fMRI data. NeuroImage, 14, 1370–1386. <http://doi.org/10.1006/nimg.2001.0931>.

Zhang, Y. Brady, M., Smith, S., 2001. Segmentation of brain MR images through a hidden Markov random field model and the expectation-maximization algorithm. IEEE. Trans. Med. Imag, 20, 45–57. <https://doi.org/10.1109/42.906424>.
